# Supplementary material for: Compliance with the WHO recommended 8+ antenatal care contacts schedule among postpartum mothers in eastern Uganda: A cross-sectional study
Source: PLoS One. 2024 Dec 9;19(12):e0314769. doi: 10.1371/journal.pone.0314769 (PMC11627358; doi:10.1371/journal.pone.0314769)
Supplement: S2 File — (PDF) [file pone.0314769.s002.pdf]

STUDY QUESTIONNAIRE

Socio-demographic characteristics

Age in years…………………………………………………………...

Highest Education of the Mother

None ☐	Primary ☐	Secondary ☐	Tertiary ☐

Highest Education of the Partner/Husband

None ☐	Primary ☐	Secondary ☐	Tertiary ☐

Marital status

Single ☐	Married ☐	Divorced/ Separated ☐	Cohabiting ☐

Occupation of the Mother

Formal employment ☐	Informal employment ☐	Unemployed ☐

Occupation of the Partner/Husband

Formal employment ☐	Informal employment ☐	Unemployed ☐

Religion

Muslim ☐	Catholic ☐	Anglican ☐
Born Again ☐	SDA ☐ (Seventh day Adventist)	Others Specify…………………………..

Residence

Rural ☐	Urban ☐

Who do you live with?

Spouse ☐	Parents ☐	Relatives ☐
Friends ☐	Others specify………	

Taking the past six (6) months, what was your average household earning per month?

1. Less than 100,000 shillings
2. Between 100,000 and 500,000 shillings

3. Between 500,000 and 1,000,000 shillings
4. Above 1,000,000 shillings

Cost of ANC services

Free ☐	Affordable ☐	Unaffordable ☐

Distance to health facility

How far is the Antenatal care clinic from your place of residence?

1. Less than 1km
2. 1-5 km
3. 6-10 km
4. morethan 10km

Mode of transport

1. By foot
2. Bicycle
3. Motorcycle
4. Car
5. Taxi
6. Other specify……………..

Decision making norms

Do you need permission first before you seek antenatal care services?

Yes ☐	No ☐

Do you request for the money needed to assist you seek antenatal care services?

Yes ☐	No ☐

Do you always need a companion before you go for antenatal care services?

Yes ☐	No ☐

Do you equally participate in the decision making process for healthcare during your pregnancy as your partner?

Yes ☐	No ☐

Do you think your partner was emotionally supportive during your pregnancy?

Yes ☐	No ☐

Do you think your opinions are well-respected in pregnancy related issues?

Yes ☐	No ☐

How many times did your partner accompany you during an ANC visit? …………

HIV status

Positive ☐	Negative ☐	Unknown ☐

Health Literacy and Exposure

Do you have access to a radio?

Yes ☐	No ☐

Do you have access to a TV?

Yes ☐	No ☐

Do you have access to newspapers?

Yes ☐	No ☐

Do you have access to internet through phones or computers?

Yes ☐	No ☐

Where do you get most of your health information from?

TV	Radio	Newspaper
Healthcare workers	Village health teams/community health volunteers	Family
Peers or neighbors	Internet	Others (specify)
Faith based organizations (place of worship)	School	

Have you been educated on the number of minimum visits for antenatal care?

Yes ☐	No ☐

If yes, by who?

Healthcare workers	Village health	Family

	teams/community health volunteers	
Peers or neighbors	Some nonprofit organization	Others (specify)
Faith based organization (places of worship)	School	

What are some benefits of attending antenatal care? (Tick all that apply)

Get information that helps me have a healthy pregnancy and healthy baby ☐	Receive services on sexually transmitted diseases testing (HIV testing) ☐	Vaccinations/Immunizations ☐
Prepare for safe delivery ☐	Receive mama kit ☐	Advice on nutrition of mother and baby ☐
Receive examinations on mother and baby's health for example ultrasound scans ☐	Basic health tests like weight, blood, or urine tests ☐	
Others Specify………………………………………………………………………………………		

In your view, at how many months should a pregnant woman first access Antenatal care services?

………………………………

How many times should a pregnant a woman receive Antenatal care services during the entire pregnancy?

……………………………………

Obstetric factors

Gravidity…………	Parity……………..	Number of living children……………………
Gestational age at		

birth…….		

Was this pregnancy planned?

Yes ☐	No ☐

How many pregnancies have you had in total? ……………………..

Current or previous obstetric complications

Yes ☐	No ☐

If yes, what type of complication………………………………….

History of illness during current pregnancy

Yes ☐	No ☐

If Yes, specify the illness……………………………….

COVID-19 Disruptions

On a scale of 1-10 (1 being very minimal and 10 being very greatly), how do you think COVID-19 affected your ability to attend Antenatal care visits?

…………………………………………..

Check on antenatal card;

Gestational age at first ANC visit in weeks…………..	Number of ANC visits……………..

Who did you see for antenatal care?

Doctor ☐	Nurse/Midwife ☐	Traditional Birth Attendant ☐
Community health worker ☐	Others Specify……………………………………………….	

Where did you go for antenatal care services?

Public hospital	Private hospital	Others specify

Do you have insurance coverage?

Yes ☐	No ☐

Quality of antenatal service delivery

As a part of your antenatal care during your pregnancy, did your provider do any of the following at least once?

1. Measure blood pressure
2. Take urine sample
3. Take blood sample
4. Listen to the baby's heartbeat
5. Do an ultrasound scan?
6. Talk with you about breastfeeding?
7. Talk with you about nutrition or immunization?
8. Teach you danger signs?
9. Vaccinate you against tetanus?
10. Gave you drugs; folic acid, fansidar, iron, etc.

On a scale of 1-10 (1 being no trust at all and 10 being completely trusting), how much did you trust your providers during antenatal care? …………………………………………..

Birth Outcomes

Maternal

Mode of delivery

1. Normal delivery
2. Caesarean section

Complications

Obstructed labor ☐	Eclampsia ☐	PPH ☐
☐	Others Specify…………………………………………………….	

Fetal

Live birth ☐	Fresh still birth ☐	Intra uterine fetal death ☐

Birth asphyxia ☐	Early neonatal death ☐	Macerated still birth ☐
Preterm ☐	☐	Low birth weight ☐
Others Specify…………………………………………………………………………………………		
