# Supplementary material for: Compliance with the WHO recommended 8+ antenatal care contacts schedule among postpartum mothers in eastern Uganda: A cross-sectional study
Source: PLoS One. 2024 Dec 9;19(12):e0314769. doi: 10.1371/journal.pone.0314769 (PMC11627358; doi:10.1371/journal.pone.0314769)
Supplement: Attachment — Submitted filename: Rebuttal letter_ANC.docx [file pone.0314769.s004.docx]

**Re; PONE-D-23-14122 Compliance with the WHO recommended 8+ antenatal care contacts schedule among postpartum mothers in eastern Uganda: A multicenter cross-sectional study**

Thank you for revising our manuscript.

Below is our point-by-point response to each comment.

Thank you.

| **Reviewer’s comment** | **Response to comment** | **Line number** |
| --- | --- | --- |
| Reviewer 1 | | |
| Introduction |  |  |
| 1. Lines 49-50, revise the 1st statement "Globally, approximately 810 mothers die daily due to preventable causes related to pregnancy and childbirth, and 99% of such deaths occur in low- and middle-income countries" to capture the updated or current contribution of maternal deaths from LMIC which is slightly lower that what is stated. | This has been updated according to the latest update from the World Health Organization website. Thank you for this observation. | 49-50 |
| 2. Same comment as above about sub-Saharan Africa contribution. Check WHO website on updated figures. | Thank you. We have also updated the sub-Saharan Africa contribution according to the most recent update on the World Health Organization website | 51-52 |
| 3. Line 68-69 statement "Further studies also show that women in Eastern Uganda were least likely to complete  at least eight or more ANC visits compared to those in the Northern, Western, and Central regions." should be referenced | Thank you for this observation and recommendation. This statement has been referenced. | 73 |
| 4. The authors should differentiate ANC visits from ANC contacts and have them defined or described in the introduction and maintain the consistency throughout the text. | The term ‘ANC visits’ have been revised to ‘ANC contacts’ to be more in line with the WHO’s use of vocabulary. Furthermore, we have included a description of what ‘ANC contact’ refers to in the introduction of the manuscript. | 65-66 |
| Methods |  |  |
| 5. What was the criteria for the selection of the four hospitals? | The selected hospitals have a high patient volume offering comprehensive ANC services to most areas within Eastern Uganda. | 95-97 |
| 6. The settings description does not mention anything about antenatal care services, numbers of women at these facilities, e.g. staffing levels, services available like ultrasound scan that attacks women for early ANC etc. | Thank you, we added this to the settings description. | 95-109 |
| 7. Why did the study exclude women without antenatal records. Since they were not too sick, self-report on the number of ANC contacts would be obtained by interview. What is the effect of excluding them? | We excluded women without ANC cards because of recall bias. With a culture of poor antenatal attendance and high illiteracy from prior knowledge in our study group, most women are less likely to precisely remember the times they attended and what was done for them per visit which we would easily ascertain on the card. We believe that we collected large enough sample of those that had cards therefore their exclusion would not significantly affect our results. | N/A |
| 8. Sample size calculation for factors associated considered age of the mothers and they obtained sample size of 1104. Was clustering taken into account and what were the assumptions | Thank you for your comment. After calculating the sample size using the formula to determining sample size for factors, clustering was taken into account. We identified the average number of deliveries at each of the four hospitals. This gave us the total sample from which we are to get from our calculated sample. The sample size per hospital was obtained by calculating; Number of deliveries per month/ Total sample population* Sample size  We have further highlighted this in the methods section. | 131-132 |
| 9. The obstetrics variables in lines 125 & 126, includes first ANC contact timing. This is included in the dependent variable. It should be excluded as independent variable. However, if they are looking for the number of ANC visits, then it is valid to include ANC timing as independent variable | Thank you for bringing up this important point. Yes, when assessing compliance, we followed the WHO schedule outlined in table 1, line 127. We categorized a woman as compliant if she had the recommended number of contacts at that specific gestational age, regardless of whether her first contact occurred within the first trimester or later. For instance, if a woman is expected to have had four contacts by 30 weeks of pregnancy and she indeed had them, irrespective of when the first one occurred, she would be considered compliant according to our approach. Therefore, having the first ANC visit within the first 12 weeks is not explicitly part of the dependent variable; therefore we treated it as an independent variable. We welcome further discussion on this matter. Thank you for your input. | N/A |
| Results |  |  |
| 10. The authors need to revise this statement "Majority had delivered from a referral hospital (n=624 56.5%)". Three four facilities (study sites) with exception of Iganga hospital were regional referrals by the government of Uganda. Re-analyze this. | We had two referral hospitals (Jinja Regional Referral Hospital, and Mbale Regional Referral Hospital) and two general hospitals (Iganga General Hospital (IGH), and Kamuli General Hospital) contrary to what you observed. We therefore think the statement has no issue unless otherwise clarified. Thank you | 94-95 |
| 11. Lines 161-162 the authors state that 196 (23.2%) had attended their first antenatal care visit within the first twelve weeks of gestation. However, table 2 shows higher number of women (total of 358). Please clarify. | Thank you for your observation, this has been clarified. | 180 |
| 12. Line 166, the first ANC visit timing were associated with outcome is misleading (see comment #9). this is part of the measure of the outcome. Should be dropped | Thank you for your comment. Yes, when assessing compliance, we followed the WHO schedule outlined in table 1, line 127. We categorized a woman as compliant if she had the recommended number of contacts at that specific gestational age, regardless of whether her first contact occurred within the first trimester or later. For instance, if a woman is expected to have had four contacts by 30 weeks of pregnancy and she indeed had them, irrespective of when the first one occurred, she would be considered compliant according to our approach. Therefore, having the first ANC visit within the first 12 weeks is not explicitly part of the dependent variable; therefore, we treated it as an independent variable. And its observed there are mothers were compliant to the schedule (had the recommended number of ANC contacts as per gestational age) but didn’t have their first ANC visit in the first 12 weeks). | N/A |
| 13. Line 167-176 is repetition of the results on table 3. consider re-writing that section | We described the results that we thought were salient from table 3 as opposed to rewriting out the entire table results, which we think should be acceptable in scientific writing. Thank you. | N/A |
| 14. The variable " where did you go for ANC" appears in the results table 3 first time (not mentioned in the methods). Please clarify. | This has been included within the independent variables list in line 142. Thanks for the observation. | 142 |
| 15. Line 196-200. I think the authors have misinterpreted this. Using a measure of recommended schedule by gestation would get fewer women meeting that schedule compared to obtaining 8 visits. | We appreciate your concern. While the frequency of ANC visits is significant, the timing of these visits is equally vital. Failing to attend during the appropriate periods may result in missing critical conditions that could have been managed more effectively and with less impact on pregnancy outcomes. Hence, we believe it's essential for women not only to meet the required number of visits but also to do so within the appropriate time frames. Thank you for your consideration. | N/A |
| 16. I proposed you include as a limitation on the exclusion of women without ANC cards. | Based on our explanation to response 7 above, we think this wouldn’t be a limitation. | N/A |
| 17. How many of these facilities had an ultrasound scan and which of them was free. | Thank you, we have clarified this in the settings description. Three of these facilities including Iganga hospital, Jinja and Mbale regional referral hospitals have an ultrasound scan which is free. | 95-109 |
| **Reviewer 2** | | |
| 1. How long the normal postnatal mothers stay in the hospital before discharge? Is it standard to stay normal labor mothers 48hrs (3days) in the hospital in Uganda? This is because you recruited postnatal mothers who had given birth within 48 hours. | Normal postnatal mothers stay in hospital on average for 24 hours. Mothers were approached before discharge, and those that consented, were recruited into the study. Some mothers were discharged earlier than 24 hours and others were discharged after 24 hours. However, all mothers were recruited before 48 hours postpartum. | N/A |
| 2. Many countries still using old WHO four ANC visits and they are not implementing eight ANC contacts. Does this eight ANC contact schedule is implemented in Uganda? When implemented? Implementation status is important before studying compliance. | The Uganda Ministry of Health has integrated an eight-contact model into the national sexual and reproductive health policy guideline. In 2018, efforts were initiated to transition towards this eight-contact ANC model. While many facilities have adopted it, the extent of its implementation hasn't been extensively studied. It's a noteworthy observation, and we will take it into account for future research recommendations. Thank you for bringing this to our attention. | 63-64 |
| 3. The measurement of dependent variable is not clear or clearly stated. When a mother compliant to the WHO recommended 8+ ANC contacts? With all 8+ ANC and appropriate gestational age? | Thank you for this comment. We categorized a mother as compliant if the number of contacts she had at a specific gestational age matched the expected visits according to the WHO 8+ contact schedule. These visits might not have occurred precisely at the exact gestational age but within the expected total by that particular gestational age. For example, by the 34th week of gestation, a mother should have had 5 contact visits. If she indeed had these five visits, she would be considered compliant, even if her first visit was at 20 weeks, followed by the third to fifth contacts between weeks 29 to 34, rather than the third visit occurring by week 26 as exactly stated in the schedule. Essentially, our formula correlated the number of contacts with gestational age rather than focusing on specific time stamps for when the ANC visits occurred. | N/A |
| 4. It is better to show a schematic presentation of the sampling procedure to clarify how the participants are selected from each hospital. | Thank you, we have clarified on the number of participants recruited from each hospital. | 133-135 |
| 5. You have to be consistent with using ‘ANC contacts’ rather than ‘ANC visits’ when you are talking about new WHO 8+ ANC model because they are quite different. | Thank you, this has been done. | N/A |
| 6. Make your tables quality tables by formatting them to be easily understandable. | In light of benchmarking from various studies, we have found that the format in which our tables are presented is quite clear and follows conventional standards. Could you please specify exactly what aspects of the table format you find unclear or problematic? This will help us better understand your concerns and make any necessary improvements. Thank you | N/A |
| 7. can you add more "discussion “to your section with research done globally in new WHO 8+ contacts instead of comparing each result with other studies done on old four ANC visit approaches. | Thank you, we revised the discussion accordingly. | 203-239 |
| 8. Please suggest a strong recommendation based on your findings. | The recommendation was included in the conclusion. Second paragraph | 292-300 |
