# Supplementary material for: Compliance with the WHO recommended 8+ antenatal care contacts schedule among postpartum mothers in eastern Uganda: A cross-sectional study
Source: PLoS One. 2024 Dec 9;19(12):e0314769. doi: 10.1371/journal.pone.0314769 (PMC11627358; doi:10.1371/journal.pone.0314769)
Supplement: Attachment — Submitted filename: Reviewer 2 Comments.docx [file pone.0314769.s005.docx]

Comments on**” Compliance with the WHO recommended 8+ antenatal care contacts schedule among postpartum mothers in eastern Uganda : A multicenter cross-sectional study”**

Overall, this is an interesting and valuable study contributes to the present literature and sharing with a wider audience.

I have not been part of the original review but having read the current version the authors have addressed the concern raised in the previous round.

I have some additional points that need to be addressed for the manuscript to be suitable for publication. Some of the issues remain and others are listed below for the revision.

- The study is trying to address area of a public health important. However, the contents are not coherent and some of the areas are not well described.
- The author used goal –oriented ANC visit and WHO recommended 8+ contacts alternatively. Goal- oriented ANC visited (Focused ANC) is the model used before the recommendation of WHO 8+ ANC contact (ANC for a positive pregnancy experience).It has to be standardized and the consistency should be maintained.
- Introduction and objectives of the study did not the operational definition given for dependent variable. The objective and the introduction section presented about compliance with WHO recommended 8+ contact and associated factors. The definition described the evaluation of compliance of the WHO 8+ recommendation against gestational age. eg. if a woman had her first visit at 34 weeks, how this will be considered in the context of the current study? Make it consistent.
- Please avoid using “didn’t”. write it as “Did not”
- Some of the results needs to presented the details to give clear picture of the findings like compliance of WHO 8+ recommendation against gestational age.

Detailed comments below (lines used from non - tracked changes version of the manuscript):

Title of the study:

- Line 1-2. The term “ Multcenter “ study for the title may not fulfil the concept of multicentre study. A multicenter study should cover different geographic areas where different languages and cultures practiced. One of its characteristics is about getting ethical clearance at different sites and data transferring across boards. The study was done in one region in eastern Uganda that covers multiple sites not multicenters. The author has change the term to the appropriate one.

Abstract:

- Line 28-29, The current WHO ANC guide line is not goal-oriented. It is called as or "Antenatal care for a positive pregnancy experience". It needs revision
- Line 42, Male involvement did not associated with Compliance of WHO 8+ recommendation as it was written in this section. The author should revise it based on the finding of the study and specify the target groups than recommend for all males.

Methods:

- Line 82-83, “This was a multi-center cross sectional study involving mainly quantitative methods of data collection”. this section needs to be revised to ensure it is clear for the case of “multicentre” study and if any other methods of data collection used rather than quantitative.
- Ine 87-91, Overall, how many referral and general hospitals found in eastern Uganda?
- Line 92-98, what sampling method was used to select those four hospitals rather than delivery load?
- Did stated method based on delivery load was used for sample size calculation or for selection of study participants using systematic method?
- Line 101-102, the author explained that some mothers were discharged before 48hrs of postpartum and the study included those who stay in the facility for 24 hours. If staying for 48 hours after delivery is not a standard in Uganda, in this case the one who stayed more might be those who had complications during either pregnancy or delivery that may enforce them to have more frequent ANC contacts than others. How selection bias was managed in this study? Did the author done any thing to check this bias?
- Line 115-116, The way the sample size calculated was not clear. The way sample size was calculated was somewhat stringent. Did sample size was calculated at the beginning for all hospitals and the calculated sample size is allocated for each hospitals using proportion to size (PPS) on the number of deliveries or it was calculated independently? If it was calculated, independently how design effect be considered and effect of clusters be managed? The author has to describe it clearly and how show sample size was calculated.
- Line 116, Did this sampling techniques is probability sampling that make the result of the study be generalized for a wider population? Use a schematic presentation of the sampling procedure to for easy understanding.
- Line 121, was it positive pregnancy experiences model? Did this definition implies for all contacts or for a single contacts? Did compliance consider frequency of contact against recommended gestational age or a contact with correct gestational age?
- Line 134, (https://www.kobotoolbox.org/),should the details not be put under References?

Results**:**

- Line 150-154, the description of in the text did not included the contents of the table. The table contains sociodemographic charterer with the status of compliance of ANC contacts. Describe it as well.
- Line 159-162, this section is the main research question addressed in this study. The frequency with gestational should be presented in detail. .Again check the title on Line 159 and definition of dependent variable given in this study to make it consistent. ANC contact Vs gestational age or contact Vs schedule based on the earlier suggestions?
- Line 169, the result of regression did not indicated association with compliance of WHO recommendation. Revise the sentence based on the results.
- Table 3, why participants attended their ANC in public and private hospitals were included? What happened to those mothers who attended at health center III?
- Line 183 – 186, was the time first ANC contact considered as dependent variable in method section? Figure 1 is not clear and self-explanatory, It is better to present it “X” axis for gestational and schedule of ANC contact and “Y” axis for proportion of mothers than present one..

Discussion

- Line 219-221, it is good not to state evidences as for example. Avoid to use such prefixes. Rather synthesize the sentence.
- Line 253-256, the sentence did not alien with the objective of the study. The objective of the study was not evaluation of ANC contacts against gestational age. It is evaluation of compliance and associated factors. Revise it accordingly.
- Line 260-262, Move this sentence to recommendation sections as a recommendation of future study
