# Supplementary material for: Compliance with the WHO recommended 8+ antenatal care contacts schedule among postpartum mothers in eastern Uganda: A cross-sectional study
Source: PLoS One. 2024 Dec 9;19(12):e0314769. doi: 10.1371/journal.pone.0314769 (PMC11627358; doi:10.1371/journal.pone.0314769)
Supplement: Attachment — Submitted filename: Response Letter_Compliance_Plos One_RN.docx [file pone.0314769.s006.docx]

| COMMENT | | **RESPONSE** | **Line number** |
| --- | --- | --- | --- |
| **EDITOR** | | |  |
| 1. | Please proofread the entire article for language correction and grammar. | - We have proofread the manuscript; thank you. | N/A |
| 2. | Table 2: Add a single space between the number and frequency in brackets. 164(19.4), it should be corrected as 164 (19.4). Please follow this rule in all tables and text. Line 184: 358 (32.4%)had, it should be 358 (32.4%) had. Please see all text and tables for this suggestion. | - Thanks for this observation; we have rectified it throughout the manuscript. | 189-190 |
| 3. | While discussing timely initiation of ANC, I suggest the authors use, consult, and add the following citation into the revised manuscript (https://www.dovepress.com/sociodemographic-predictors-of-initiating-antenatal-care-visits-by-pre-peer-reviewed-fulltext-article-IJWH). | - Thank you for suggesting the article. We have used information about predictors of timely ANC initiation from this article in our discussion, and it was helpful. | 254-258 |
| 4. | Discussion needs a bit work. The authors should add recommendations at the end of each paragraph from each of the key observation they have made. | - We have provided necessary recommendations for our key observations. Thank you | 220-297 |
| **REVIEWER 3** | | |  |
|  | **General Comments** | |  |
| 5. | The author used goal –oriented ANC visit and WHO recommended 8+ contacts alternatively. Goal- oriented ANC visited (Focused ANC) is the model used before the recommendation of WHO 8+ ANC contact (ANC for a positive pregnancy experience). It has to be standardized and the consistency should be maintained. | - This has been harmonized throughout the manuscript, thanks for noting that discrepancy and clarification. | N/A |
| 6. | Introduction and objectives of the study did not the operational definition given for dependent variable. The objective and the introduction section presented about compliance with WHO recommended 8+ contact and associated factors. The definition described the evaluation of compliance of the WHO 8+ recommendation against gestational age. eg. if a woman had her first visit at 34 weeks, how this will be considered in the context of the current study? Make it consistent. | - To clarify why we used this approach and its pragmatism. We have added a detailed explanation to study variable sections with an example to give the reader the reason behind this approach. We hope that this can provide more clarity. - We have added a statement to the study's objective in the introduction to contextualize the definition of our dependent variable. | 135 to 138 |
| 7. | Please avoid using “didn’t”. write it as “Did not” | - This has been rectified, thank you. | 181 |
| 8. | Some of the results needs to presented the details to give clear picture of the findings like compliance of WHO 8+ recommendation against gestational age. | - We think now with a clear description of our approach in the methods, the reader should be able to appropriately look at the results as presented. Thank you | 173-215 |
| 9. | Line 1-2. The term “ Multcenter “ study for the title may not fulfil the concept of multicentre study. A multicenter study should cover different geographic areas where different languages and cultures practiced. One of its characteristics is about getting ethical clearance at different sites and data transferring across boards. The study was done in one region in eastern Uganda that covers multiple sites not multicenters. The author has change the term to the appropriate one. | - We have forfeited the use of the word multicenter in our title. Thank you. | 1-2 |
|  | **Abstract** | |  |
| 10. | Line 28-29, The current WHO ANC guide line is not goal-oriented. It is called as or "Antenatal care for a positive pregnancy experience". It needs revision | - This has been rectified; thanks for noting that discrepancy. | 28-29 |
| 11. | Line 42, Male involvement did not associated with Compliance of WHO 8+ recommendation as it was written in this section. The author should revise it based on the finding of the study and specify the target groups than recommend for all males. | - Indeed, the statement regarding partner involvement was general and unclear, not truly representative of the findings. We have completely restructured the conclusion to align with the study findings. | 41-44 |
|  | **Methods** | |  |
| 12. | Line 82-83, “This was a multi-center cross sectional study involving mainly quantitative methods of data collection”. this section needs to be revised to ensure it is clear for the case of “multicentre” study and if any other methods of data collection used rather than quantitative. | - The study design section has been edited for grammatical clarity. We have foregone the use of ‘multicenter’ based on your guidance that doesn’t meet its use and also clarified that this wasn’t just ‘mainly’ but entirely quantitative. | 85 |
| 13. | Ine 87-91, Overall, how many referral and general hospitals found in eastern Uganda? | There are three regional referral hospitals and six general hospitals in eastern Uganda | 91-93 |
| 14. here | Line 92-98, what sampling method was used to select those four hospitals rather than delivery load? | These hospitals were purposively selected based on the delivery load. | 93 |
| 15. | Did stated method based on delivery load was used for sample size calculation or for selection of study participants using systematic method? | We used proportionate to size sampling to estimate the sample size for each hospital and participants at each hospital were recruited by consecutive sampling. | 125-126 |
| 16. | Line 101-102, the author explained that some mothers were discharged before 48hrs of postpartum and the study included those who stay in the facility for 24 hours. If staying for 48 hours after delivery is not a standard in Uganda, in this case the one who stayed more might be those who had complications during either pregnancy or delivery that may enforce them to have more frequent ANC contacts than others. How selection bias was managed in this study? Did the author done any thing to check this bias? | All mothers including those discharged before 48 hours were enrolled into study thus selection bias was unlikely. |  |
| 17. | - Line 115-116, The way the sample size calculated was not clear. The way sample size was calculated was somewhat stringent. Did sample size was calculated at the beginning for all hospitals and the calculated sample size is allocated for each hospitals using proportion to size (PPS) on the number of deliveries or it was calculated independently? If it was calculated, independently how design effect be considered and effect of clusters be managed? The author has to describe it clearly and how show sample size was calculated. | Thank you, we have revised this as guided. | 125-127 |
| 18. | Line 116, Did this sampling techniques is probability sampling that make the result of the study be generalized for a wider population? Use a schematic presentation of the sampling procedure to for easy understanding. | Thank you, we have added a schematic presentation of the sampling procedure. | 129-130 |
| 19. | Line 121, was it positive pregnancy experiences model? Did this definition implies for all contacts or for a single contacts? Did compliance consider frequency of contact against recommended gestational age or a contact with correct gestational age? | We have revised this, thank you. | 135 |
| 20. | Line 134, (https://www.kobotoolbox.org/),should the details not be put under References? | Thank you, we added this to the references as suggested |  |
|  | **Results** | |  |
| 21. | Line 150-154, the description of in the text did not include the contents of the table. The table contains sociodemographic charterer with the status of compliance of ANC contacts. Describe it as well | - We have described that part of the results as well. Thank you. | 181-186 |
| 22. | Line 159-162, this section is the main research question addressed in this study. The frequency with gestational should be presented in detail. Again check the title on Line 159 and definition of dependent variable given in this study to make it consistent. ANC contact Vs gestational age or contact Vs schedule based on the earlier suggestions? | - Thank you for your feedback. However, we would like to clarify the study's methodology. Our approach did not simply involve counting ANC visits, but rather evaluating them in relation to gestational age at delivery, which provides a more objective measure. As explained in the methods section, a woman delivering at 36 weeks with 6 contacts would still be on track with the recommended schedule, even though she hadn’t reached 8 visits. This is why we used this method to assess compliance with the "8+ ANC contact schedule" during analysis. At the stage of presenting results, our focus is on reporting on compliance and the factors associated. We don’t see the need again to drag the frequency of visits used in calculating the compliance into the results section. We hope this clears up any confusion |  |
| 23. | Line 169, the result of regression did not indicated association with compliance of WHO recommendation. Revise the sentence based on the results. | - Based on our analysis, “attendance of the first antenatal care contact within 12 weeks of gestation [AOR: 6.42; 95% CI: (4.43 to 9.33)], having 2 to 4 children [AOR: 0.65; 95% CI: (0.44 to 0.94)], having an unemployed spouse [AOR: 1.71; 95% CI: (0.53 to1.08)] and having insurance coverage were associated with compliance. | 189-200 |
| 24. | Table 3, why participants attended their ANC in public and private hospitals were included? What happened to those mothers who attended at health center III? | - Participants included in this study delivered at a public hospital, however, we asked them “Where they received ANC services from?” where some reported a public hospital and others a private hospital. If a mother mentioned a health center III, it was a denoted as a public or private hospital accordingly. | N/A |
| 25. | Line 183 – 186, was the time first ANC contact considered as dependent variable in method section? Figure 1 is not clear and self-explanatory, It is better to present it “X” axis for gestational and schedule of ANC contact and “Y” axis for proportion of mothers than present one. | - First ANC visit wasn’t considered as a dependent variable, however we decided to graphically represent it here for its importance in the subject matter of compliance. - The variables gestation age and contacts per schedule helped us create an objective composite variable—compliance—which is our focus for this paper. That was a means to determine our outcome of interest. | N/A |
| 26. | **Discussion** | |  |
| 27. | Line 219-221, it is good not to state evidences as for example. Avoid to use such prefixes. Rather synthesize the sentence. | - This has been rectified. Thank you for that guidance. |  |
| 28. | Line 253-256, the sentence did not alien with the objective of the study. The objective of the study was not evaluation of ANC contacts against gestational age. It is evaluation of compliance and associated factors. Revise it accordingly. | - We have modified the statement. Although it also meant to provide a probable reason for the differences from a methodological point of view. |  |
| 29. | Line 260-262, Move this sentence to recommendation sections as a recommendation of future study | - This has been done, thank you. |  |
